# Supplementary material for: Comprehensive Annotation and Functional Exploration of MicroRNAs in Lettuce
Source: Front Plant Sci. 2021 Dec 24;12:781836. doi: 10.3389/fpls.2021.781836 (PMC8739914; doi:10.3389/fpls.2021.781836)
Supplement: Supplementary file 2 [file Data_Sheet_8.ZIP › GSM2230747.plot/Lsa-miRN40_Lsat_1_v5_gn_3_103621.1_1294_TPlot.pdf]

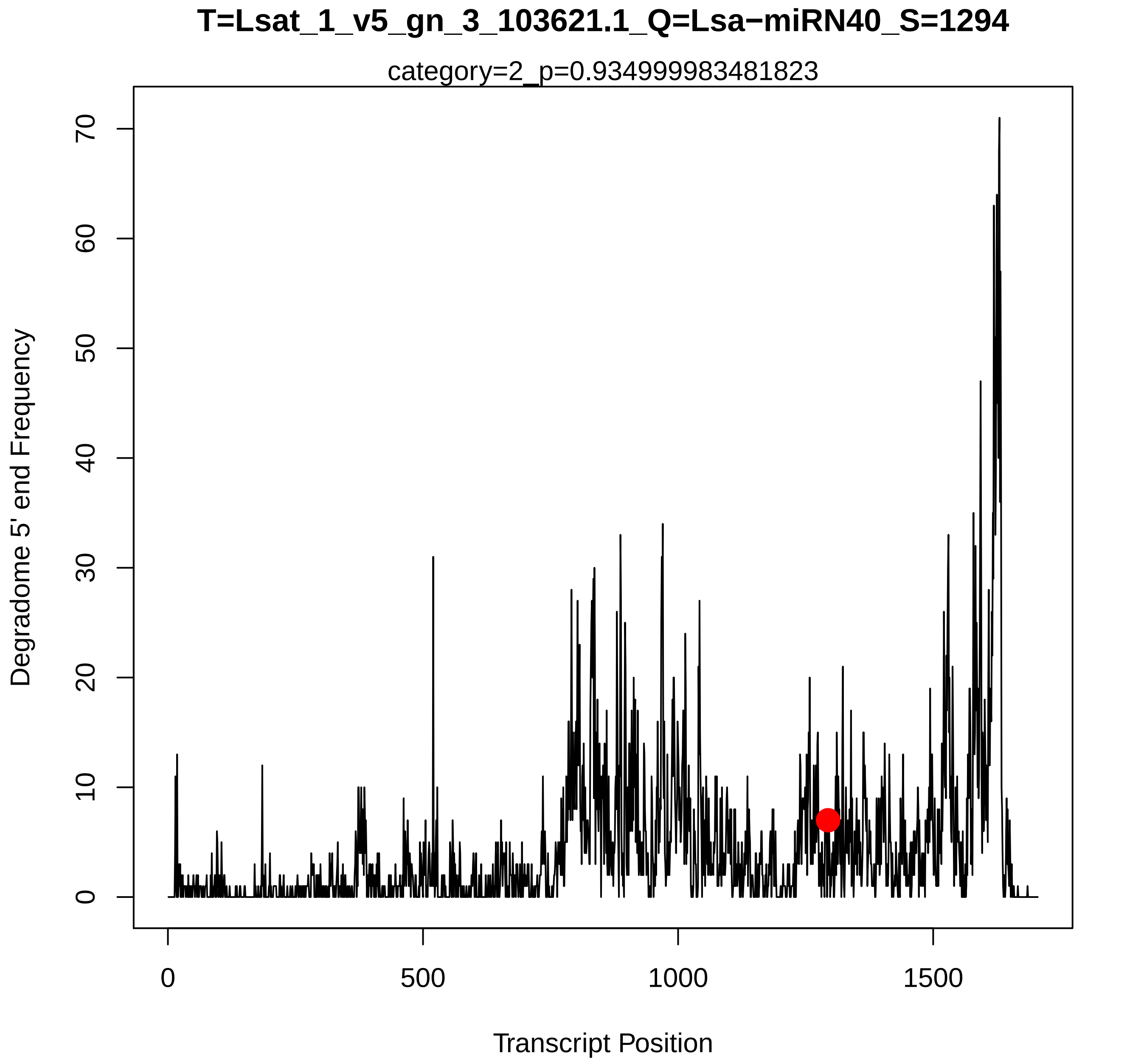
T=Lsat_1_v5_gn_3_103621.1_Q=Lsa−miRN40_S=1294
category=2_p=0.934999983481823
Degradome 5' end Frequency
0
10
20
30
40
50
60
70
0
500
1000
1500
Transcript Position
